# Tunable Supramolecular Chirogenesis in the Self-Assembling of Amphiphilic Porphyrin Triggered by Chiral Amines

**DOI:** 10.3390/ijms21228557

**Published:** 2020-11-13

**Authors:** Marco Savioli, Manuela Stefanelli, Gabriele Magna, Francesca Zurlo, Maria Federica Caso, Rita Cimino, Claudio Goletti, Mariano Venanzi, Corrado Di Natale, Roberto Paolesse, Donato Monti

**Affiliations:** 1 Department of Science and Chemical Technology, University Tor Vergata, Via della Ricerca Scientifica 1, 00133 Rome, Italy; svlmrc01@uniroma2.it (M.S.); manuela.stefanelli@uniroma2.it (M.S.); magna.gabriele@uniroma2.it (G.M.); francesca.zurlo@uniroma2.it (F.Z.); rita.cimino@uniroma2.it (R.C.); venanzi@uniroma2.it (M.V.); roberto.paolesse@uniroma2.it (R.P.); 2 ENEA, Centro Ricerche Frascati, Via Enrico Fermi 45, 00044 Frascati, Italy; mariafederica.caso@enea.it; 3 Department of Physics, University Tor Vergata, Via della Ricerca Scientifica 1, 00133 Rome, Italy; goletti@roma2.infn.it; 4 Department of Electronic Engineering, University Tor Vergata, Via del Politecnico 1, 00133 Rome, Italy; dinatale@uniroma2.it; 5 Department of Chemistry, University La Sapienza, P.le A. Moro 5, 00185 Rome, Italy

**Keywords:** porphyrinoids, supramolecular chemistry, chirality

## Abstract

Supramolecular chirality is one of the most important issues in different branches of science and technology, as stereoselective molecular recognition, catalysis, and sensors. In this paper, we report on the self-assembly of amphiphilic porphyrin derivatives possessing a chiral information on the periphery of the macrocycle (i.e., *D*- or *L*-proline moieties), in the presence of chiral amines as co-solute, such as chiral benzylamine derivatives. The aggregation process, steered by hydrophobic effect, has been studied in aqueous solvent mixtures by combined spectroscopic and topographic techniques. The results obtained pointed out a dramatic effect of these ligands on the morphology and on the supramolecular chirality of the final self-assembled structures. Scanning electron microscopy topography, as well as fluorescence microscopy studies revealed the formation of rod-like structures of micrometric size, different from the fractal structures formerly observed when the self-assembly process is carried out in the absence of chiral amine co-solutes. On the other hand, comparative experiments with an achiral porphyrin analogue strongly suggested that the presence of the prolinate moiety is mandatory for the achievement of the observed highly organized suprastructures. The results obtained would be of importance for unraveling the intimate mechanisms operating in the selection of the homochirality, and for the preparation of sensitive materials for the detection of chiral analytes, with tunable stereoselectivity and morphology.

## 1. Introduction

**Chart 1:**
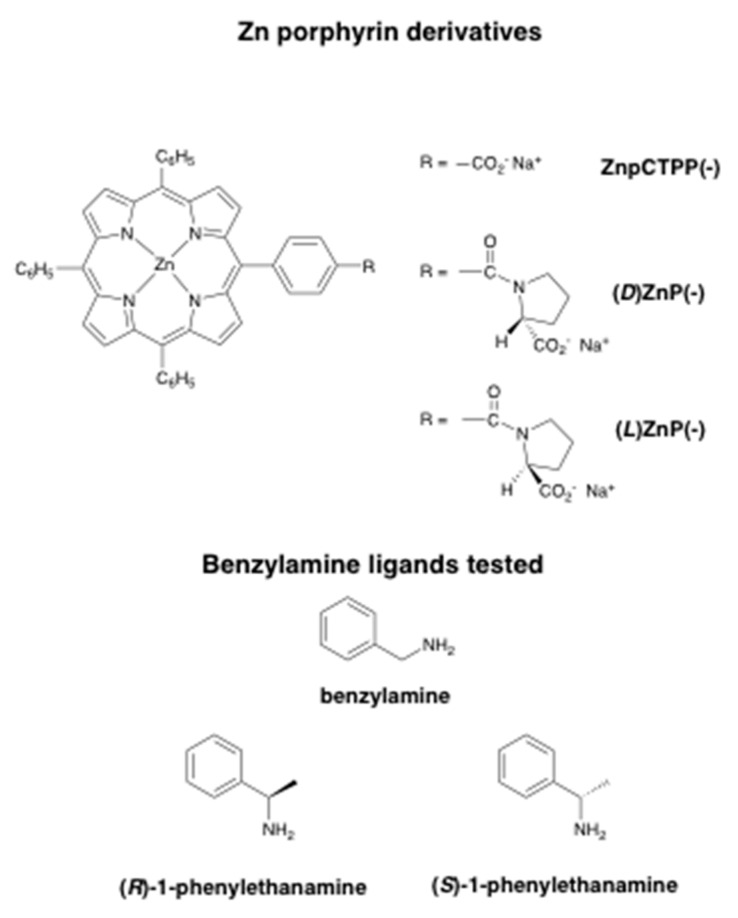
Molecular structures of porphyrin and benzylamine derivatives studied in this work.

Porphyrin-based supramolecular structures [1] are of fundamental importance in vast areas of science and technology as, for example, photosensitizers in photodynamic therapy of tumors (PDT) [2], nonlinear optics [3], sensors field [4,5], and nanotechnology [6]. The importance and the applicability of these structures could be even more widened by the introduction of elements of chirality [7] for the construction, among others, of stereoselective sensors able to detect selectively the different enantiomers of natural products, or their derivatives [8,9]. In addition to the fundamental role of the stochastic “spontaneous symmetry breaking”, which would have been involved in the selection of homochirality in biological systems [10], the construction of porphyrin aggregates with specific chirality could be achieved by two main strategies, relying on (a) self-assembling of achiral macrocycles upon the action of external effectors and (b) self-assembling of intrinsically chiral building blocks in controlled specific conditions. As far as the first issue is concerned, chiral suprastructures of achiral tetrapyrrolic macrocycles could be obtained by the effect of external directional forces such as stirring [11,12], magnetic fields [13], Langmuir–Blodgett or Langmuir–Schafer techniques [14], chiral co-solutes [15,16], chiral templates [17,18] or, very recently, by circularly polarized light [19]. In some cases, the effect of adventitious traces of chiral contaminants has also been reported [20,21]. The second approach relies on the presence on the macrocycles periphery of stereochemical information, which can be effectively read-out and transferred at supramolecular level during the self-assembly process. Along this line, we were interested in the study of the aggregation process of amphiphilic tetraphenylporphyrin derivatives, functionalized by chiral polar or ionic functionalities such as sugars [22,23,24], steroids [25,26,27,28] or charged proline derivatives [29,30,31], acting as stereochemical information to be recognized and efficiently transmitted during the stereospecific formation of the final supramolecular mesoscopic structures. Very recently, we investigated the self-assembly behavior of intrinsically chiral zinc-tetraphenylporphyrin derivatives, characterized by the presence of covalently linked enantiomers of anionic *L*- or *D*-prolinate groups on the molecular frame [32] (Chart 1).

Spectroscopic and kinetic studies revealed that the self-assembly process followed a biphasic path, in which a first fast formation of aggregation nuclei is followed by a slow autocatalytic step, to give chiral fractal mesoscopic structures of micrometric size with high degree of stereospecificity, strictly depending on the (*L*)- or (*D*)-configuration of the appended moiety. We surmised that the stereospecificity of the process is the result of the coordination of the carboxylate group of a proline moiety to the Zn(II) ion of a neighboring macrocycles, resulting in a final structure featuring strong supramolecular chirality. The important role of ligand to metal coordination in the formation of chiral porphyrin-based supramolecular species [33,34], as well as other structures [35], has been already demonstrated. Moreover, coordination of chiral amine or similar donor molecules to Zn-porphyrin derivatives is a well-known issue, exploited for the molecular recognition of chiral analytes and the determination of their absolute configurations [36,37].

In this work, we report on the effect of the presence of either chiral or achiral external nitrogen ligands on the aggregation process of (*L*)ZnP(-) and (*D*)ZnP(-) porphyrin macrocycles, carried out in an aqueous solvent mixture. The results have been compared to those obtained from analogous experiments carried out on an achiral porphyrin analogue (ZnpCTPP(-), Chart 1), evidencing not only the effect of the coordination of the external ligands to the central metal ion on both the final supramolecular chirality, and the morphology of the final species, but also the importance of the proline residues for the achievement of the whole highly organized supramolecular structures. The results obtained, besides their intrinsic value from an academic point of view, are of importance for the construction of supramolecular species with defined morphology, as a consequence of the tuning of subtle interplay of interactions.

## 2. Results and Discussion

### 2.1. Aggregation Studies

Aggregation of porphyrins (*L*)- and (*D*)ZnP(-) has been carried out in hydroalcoholic solutions (EtOH/H_2_O 25:75 *v*:*v*; 298 K) at 5 μM and 10 μM concentrations by strictly following a “porphyrin first” protocol, and under the effect of sonication, in the presence of a 1:100 molar ratio of (*R*)- or (*S*)-1-phenylethanamine. Benzylamine has also been employed as achiral counterpart (Chart 1). This procedure, thoroughly described in the Experimental section, allowed for a good reproducibility of the results and a convenient reaction time, to be followed by conventional spectroscopic techniques.

**Scheme 1:**
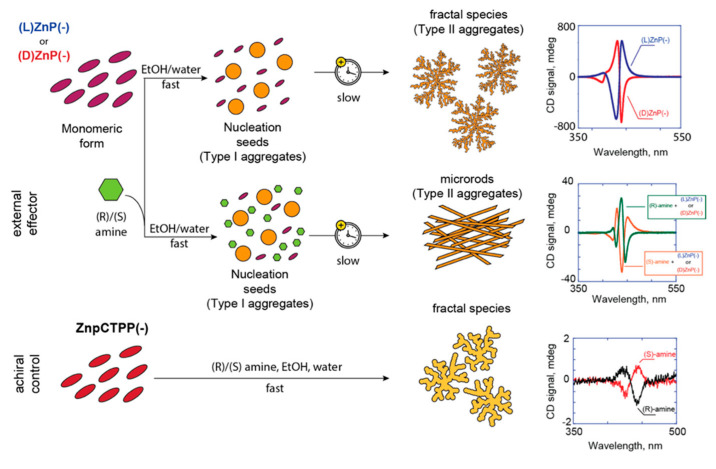
Pictorial representation of the aggregation path of chiral porphyrins ((*L*)- and (*D*)ZnP(-)) in the absence or in the presence of external chiral amines, and comparison with the achiral macrocycle (ZnpCTPP(-)).

According to the experimental results, we may anticipate that (i) the process occurs through two distinct steps, characterized by an initial fast nucleation completed within the time of sample preparation (Type-I aggregates; Scheme 1), which is followed by a slower evolution toward the formation of highly organized structures featuring intense supramolecular chirality, as shown by circular dichroism spectroscopy (Type-II aggregates; Scheme 1); (ii) the final configuration of these species is dictated by the stereochemistry of the external ligands, disregarding that of the chiral appended group of the tetrapyrrolic macrocycles, (iii) the final structures feature rod-like morphology, different from the fractal species obtained in the absence of external ligand, and (iv) the intensities of the Circular Dichroism (CD) features (i.e., the final supramolecular chirality) depend on the presence of the proline residue.

**Figure 1:**
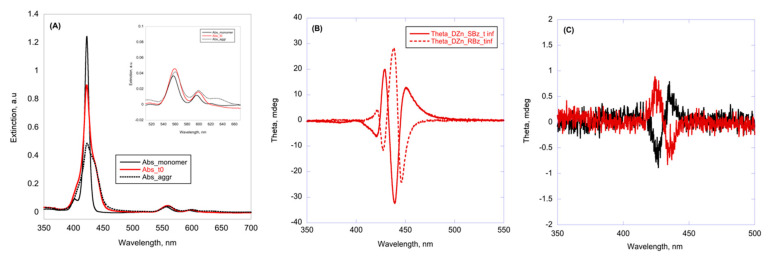
(**A**) UV-Vis spectra of (*D*)ZnP(-) in the presence of (*S*)-1-phenylethanamine: in the non-aggregative conditions (EtOH/H_2_O 50% *v*:*v*; black continuous trace; ×0.5); in EtOH/H_2_O 25/75% *v*:*v*, at time = 0 (red trace); in EtOH/H_2_O 25/75% *v*:*v*, at equilibrium (t = infinite), black dotted trace. The inset reports the variations of the corresponding Q bands. (**B**) CD spectra at equilibrium (t = infinite; EtOH/H_2_O 25/75% *v*:*v*) of (*D*)ZnP(-) in the presence of (*R*)-1-phenylethanamine, red dotted trace, and (*S*)-1-phenylethanamine, red continuous trace. (**C**) CD spectral of Type-I aggregates (5.0 μM; EtOH/H_2_O 25:75 *v*:*v*) of (*L*)ZnP(-) (red trace) and (*D*)ZnP(-) (black trace) in the presence of (*S*)-1-phenylethanamine 5.0 × 10^−4^ M. Superimposable traces are obtained in the presence of (*R*)-1-phenylethanamine.

UV-Vis and CD spectroscopic studies gave insights on the observed phenomenon. At the 5 μM concentration and in the presence of a 1:100 chiral porphyrin/chiral amine molar ratio, the first step is characterized by a fast aspecific hypochromic effect of the Soret band of the macrocycle. CD spectroscopy showed very weak coupled bands, indicating a low degree of stereospecificity of this stage. A further slow evolution, with the appearance of a red-shifted shoulder indicates the formation of J-type aggregates (Figure 1A), along with the emergence of a complex spectral pattern of the corresponding CD plots (Figure 1B), constituted by a set of two features, centered at ca 423 and at 440 nm, with that at longer wavelength of higher intensity. This characteristic pattern, whose crossover points are reported in Table S1 of Supplementary Information, has been ascribed to the formation of J-type aggregates of complex morphology, which are excitonically coupled along two preferential space directions, referred as *B_H_* and *B_J_* transitions for the features at higher and lower energy, respectively [38].

As far as the higher intensity feature is concerned, the final configuration of the aggregates (i.e., the sign of the coupled bands) is dictated by the stereochemistry of the external amine ligands, disregarding that of the chiral appended group of the tetrapyrrolic macrocycles, as shown by the corresponding CD spectra (Figure 1B; Figure S1 in Supplementary Information). In particular, as far as the *B_J_* features are concerned, the aggregates obtained in the presence of (*R*)-1-phenylethanamine featured a −/+/− pattern, whereas in the case of (*S*)-1-phenylethanamine, a mirrored +/−/+ pattern is observed. Similar pattern profiles have been reported by Monsù Scolaro, in the case of chiral templated aggregation of water-soluble porphyrin in confined environment [39]. The clear inversion of the spectral patterns of Figure 1B safely rules out the effect of adventitious chiral contaminants that could be present in solution [20,21]. 

Chosen as one of external chiral amine, the intensity of the CD spectral features are similar, within the experimental errors, for both the (*L*)ZnP(-) and (*D*)ZnP(-) confirming the overwhelming effect of the stereochemistry of the nitrogen ligand. It is known, in fact, that the binding affinity of zinc-tetraarylporphyrin derivatives to external ligands correlates well with the basicity of the donor atom, following the general order P; S < O < N [40]. In particular, benzylamine derivatives should feature much higher affinity with respect to the proline carboxylate group, the pK_a_ of their neutral form (298 K in water) being 9.3 and 1.95, respectively.

It is important to note that independent binding experiments, carried out in non-aggregative conditions (i.e., 50% EtOH/H_2_O (*v*:*v*) media) gave no evidence of the occurrence of amine coordination to the central metal ion, even at 1:1000 porphyrin/amine molar ratio due to competition by the donor solvents. This finding indicates that the driving force of the aggregation process is the result of a fine balance of synergic interactions, such as (i) coordination, (ii) hydrophobic effect and (iii) π−π interactions between the phenyl group of the benzylamines and the aromatic porphyrin platforms. During their formation, the Type-I aggregates evidently create a hydrophobic cavity, in which the binding competition of the donor solvents is hampered. This is strongly suggested by the fact that the initial oligomeric structures feature CD coupled bands of same sign and alike intensities of that obtained in the absence of chiral external amine (i.e., +/− for (*D*)ZnP(-), and −/+ in the case of (*L*)ZnP(-); Figure 1C), and that their signs (i.e., chirality) are independent of that of the external chiral nitrogen base. The need of the presence of a hydrophobic environment is further confirmed by the experimental evidence (UV-Vis and CD spectroscopy) that the pre-formed Type-II aggregates of both (*D*)- and (*L*)ZnP(-) are stable in the presence of a strong excess of phenylethanamines, as indicated by the constant intensities of the CD features over time. Analogously, the co-formed structures (i.e., porphyrin@phenylethanamines) are inert toward the addition of an excess of the amine counterpart of opposite stereochemistry.

In the case of the aggregation carried out in the presence of a 1:100 molar ratio of benzylamine, the achiral analogue, the resulting supramolecular species are CD silent, confirming the effect of the coordination to the Zn(II) ion. Both porphyrin aggregation and amine coordination have been proven by the red shift and broadening of the UV-Vis bands (Figure S2A,B Supplementary Information).

**Figure 2:**
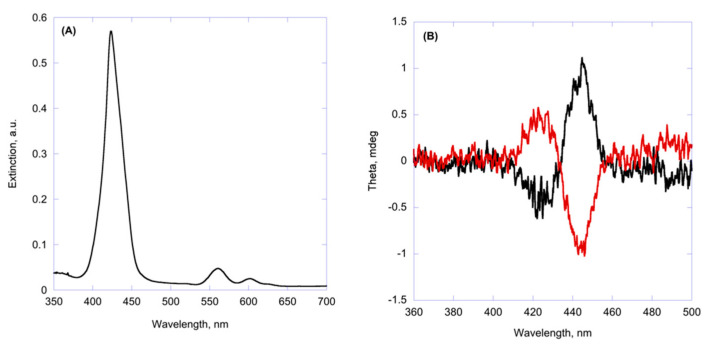
(**A**) UV-Vis of ZnpCTPP(-) 5.0 μM aggregates (EtOH/H_2_O 25:75 *v*:*v*; 298 K) in the presence of (*S*)-1-phenylethanamine 5.0 × 10^−4^ M. The spectrum obtained in the presence of (*R*)-1-phenylethanamine is virtually superimposable. (**B**) CD spectra of ZnpCTPP(-) 5.0 μM (EtOH/H_2_O 25:75 *v*:*v*; 298 K) in the presence of (*S*)-1-phenylethanamine 5.0 × 10^−4^ M (black trace); (*R*)-1-phenylethanamine 5.0 × 10^−4^ M (red trace).

The co-aggregation experiments have been also carried out on the achiral Zn-porphyrin analogue (ZnpCTPP(-); Chart 1) in order to prove the induction effect of the metal coordination by the chiral phenylethanamines. In this case, UV-Vis results showed a fast formation of aggregated structures (Figure 2A); alongside, the corresponding CD spectra showed the formation of weak bisignated coupled features, whose signs are dependent on the stereochemistry of the added chiral inductor, namely +/− with the (*S*)-1-phenylethanamine, and −/+ with the (*R*)-1-phenylethanamine (Figure 2B).

Quite surprisingly, both the UV-Vis and CD band intensities did not feature further evolution with time, indicating that the proline residue should play a striking role for the formation of more complex and structured species such as the ones described above. Finally, aggregates of ZnpCTPP(-) obtained in the absence of chiral dopants resulted in CD silent spectra over the temporal window considered, definitely ruling out the effect of adventitious chiral impurities accidentally present in solution (results not shown).

### 2.2. Kinetic Studies

Kinetic studies have been carried out in the usual solvent mixture at 298 K by following the variation of the spectral intensities (UV-Vis and CD) with time. The concentrations used are 5.0 × 10^−6^ and 5.0 × 10^−4^ M, for the porphyrins and the chiral amine ligands, respectively. As briefly introduced in the former section, in all of the cases examined the process is carried out via a biphasic mechanism, in which a fast nucleation phase is followed by a slower process of formation of the final chiral suprastructures, completed within one week. In the first step, formation of oligo-aggregated structures occurred (Type-I species), that act as nucleation seeds for the binding of both further porphyrin monomers and phenylethanamines.

**Figure 3:**
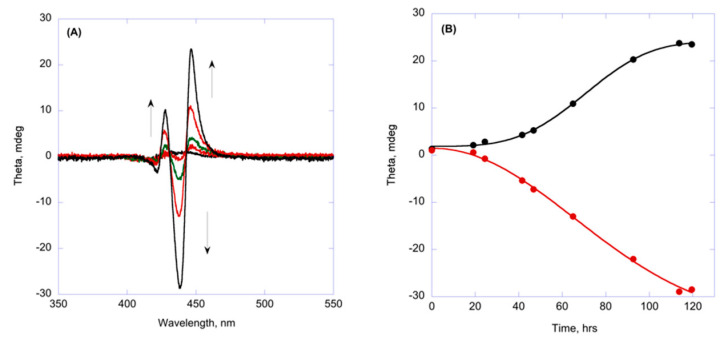
(**A**) CD spectral changes (Type-II aggregates evolution) with time of (*L*)ZnP(-) 5 μM (EtOH/H_2_O 25:75 *v*:*v*) in the presence of (*S*)-1-phenylethanamine 5.0 × 10^−4^ M. (**B**) Corresponding calculated fit (Equation (1)) at the wavelength maxima of the lower energy bands: λ = 446 nm (black circles); λ = 438 nm (red circles).

The analysis of the second step (Type-II aggregate formation) gave kinetic plots (i.e., Extinction or Theta molar vs. time) that showed a typical sigmoidal behavior, indicating the cooperative formation of the final suprastructures The traces are reported in Figure 3A,B, for the case of (*L*)ZnP(-)@(*S*)-1-phenylethanamine.

The experimental data could be successfully fitted by the Equation (1), employed in the kinetic analysis of J-aggregation of related macrocycles [41]. The equation used is of the form:

(1) $$\left[Y\right]={\left[Y\right]}_{eq}+{\left([Y{]}_{o}-[Y\right]}_{eq})e^{\left[\frac{-{\left(kt\right)}^{n+1}}{\left(n+1\right)}\right]}$$

In this equation, *[Y]*, *[Y]*_0_ and *[Y]_eq_* are the physical quantities related to the concentrations of the monomers or the aggregates, at time = t, time = 0, and at equilibrium, respectively; *n* is the “aggregate growth rate” parameter, and *k* the kinetic pseudo first-order rate constant. The results are graphically reported in Figure 3B for the experiment carried out by CD spectroscopy. Remarkably, the close adherence of the calculated curve to the experimental data can be emphasized, indicating the validity of the proposed mechanism. The calculated kinetic constant values (CD spectroscopy) are very similar for all the combinations of porphyrins and chiral amines. Their mean values are *k* = 2.8 (± 0.4) × 10^−4^ min^−1^ and *n* = 1.7 (± 0.2), confirming that the process leading to the final structures become increasingly favored with time, i.e., on increasing the size of catalytic surfaces. Very similar results have been obtained by following the corresponding extinction decay of the Soret band with time (i.e., *k* = 2.4 (± 0.3) × 10^−4^ min^−1^ and *n* = 1.5 (± 0.4); see Figure S3, in Supplementary Information). A comparison with the rate constant obtained for the process carried out in the absence of amine (*k* = 9.3 × 10^−5^ min^−1^) indicates an accelerating effect, within one order of magnitude, exerted by the added amine co-ligand [29]. Conversely, aggregation of the analogous achiral counterpart ZnpCTPP(-) resulted in a fast aggregation step, virtually completed within the time of samples preparation, both in the absence and in the presence of external co-ligands.

**Figure 4:**
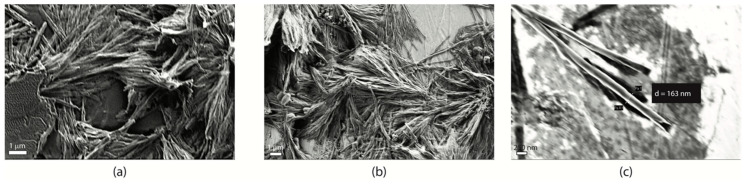
SEM topographies of precipitates from equilibrium solutions of (*D*)ZnP(-) (**a**) and (*L*)ZnP(-) 10 μM (**b**,**c**) in the presence of (*R*)-1-phenylethanamine. Images obtained with (*S*)-1-phenylethanamine are reported in Figure S5 of Supplementary information.

Analogous experiments were carried out at 10 μM and 1.0 mM of (*D*)- and (*L*)ZnP(-), and amines, respectively. Serendipitously, due to the increased concentration of the substrates, precipitation of porphyrinic material occurs on going to the completion of the process, accompanied by a corresponding strong decrease of both UV-Vis and CD features (Figure S4, in Supplementary Information). This material could be carefully collected and placed onto an aluminum stub surface for an SEM topographic investigation, or drop casted onto a microscope glass slide, for a fluorescence or fluorescence microscopy analysis. Both techniques showed the formation of peculiar structures, in the form of dense bundles of weed-like nanorod-type species with about 10 μm in length, and ca 150–200 nm width (Figure 4a–c), strikingly different from those obtained for the aggregation carried out without chiral amine dopants [32]. 

**Figure 5:**
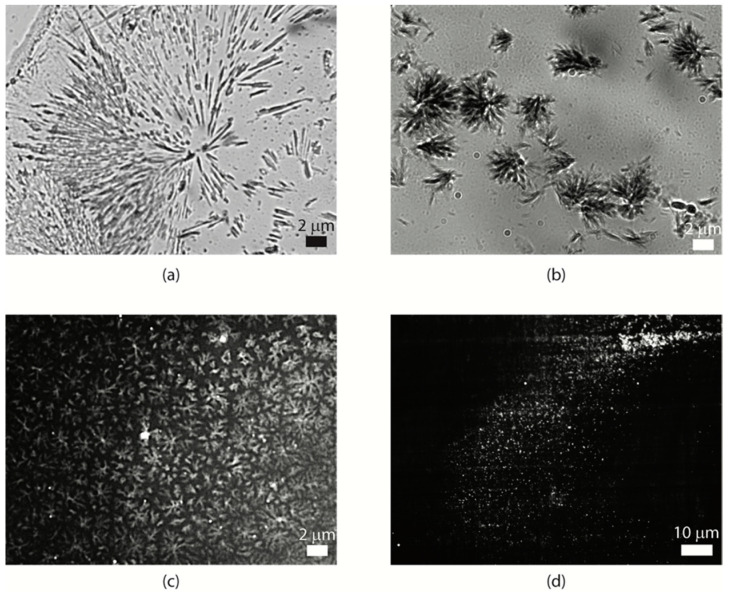
Microscope transmission images of drop casted equilibrium solution on glass of (**a**) (*D*)ZnP(-)@(*R*)-1-phenylethanamine solution; (**b**) freshly drop casted equilibrium solution with wet precipitate (see text). Fluorescence emission image of drop casted equilibrium solution of (**c**) ZnpCTPP(-)@(*R*)-1-phenylethanamine, and (**d**) ZnpCTPP(-) aggregates.

The investigation by fluorescence microscopy revealed the presence of the same structures, with strong quenching of the porphyrin emission (Figure S6, Supplementary Information), once again ascribable to amine coordination (Figure 5a,b).

As far as the aggregates formed by the achiral macrocycle are concerned, the drop casted films showed the formation of densely packed fractal structures triggered by the presence of chiral amines, whereas in the absence of chiral ligands only non-specific globular forms could be observed (Figure 5c,d), as it could be also surmised by the lack of CD features of the corresponding solutions.

An exact evaluation of the porphyrin/amine ratio in these structures, that could have given clearer information on the role of nitrogen-based ligands, could not be obtained. This due to the fact that extensive washings of the solids gave unavoidable resolubilization of the macrocycles in monomeric form (see Experimental section). 

## 3. Conclusions and Perspectives

The results obtained for the solvent-promoted aggregation of intrinsically chiral Zn-porphyrin derivatives bearing a prolinate moiety (i.e., (*L*)ZnP(-) and (*D*)ZnP(-); Chart 1) in the presence of chiral phenylethanamines indicated that the process is carried out through a complex mechanism, in which an initial rapid chaotic stage is followed by a slower autocatalytic step leading to the formation of highly organized species, in the shape of rod-like structures of micrometric size. The obtained assemblies feature intense supramolecular chirality with CD bands of complex pattern, with stereochemistry strictly depending on the stereoisomer used as chiral external dopant, as an effect of coordination to the Zn(II) central ion. However, the results obtained in the case of an analogous achiral Zn-tetrapyrrolic macrocycle (i.e., ZnpCTPP(-); Chart 1), showed the formation of fractal species through a rapid aggregation step. The chirality of the self-assembled structures still depends on the stereochemistry of the amine present in solution, but with CD bands of reduced intensities of about one order of magnitude, and of simple bisignated pattern. Finally, it is important to mention, as reported in our previous studies [32], that the aggregation of (*L*)ZnP(-) and (*D*)ZnP(-) carried out without chiral amines proceeds through alike biphasic autocatalytic mechanism, leading to final architectures featuring intense supramolecular chirality, depending on the stereochemistry of the proline appended functionality. 

The overall results pointed out that the efficiency of the transmission of the chiral information from molecular to mesoscopic scale, and of the tuning of the morphology of the final suprastructures relies not only on the coordination of ligands to the porphyrins central metal ion (i.e., either external or internal ligands), but also on the onset of subtle synergic interplay of interactions arising from, in this case, the prolinate residue. The full comprehension of the intimate nature of the interactions onset within these complex systems will be the focus of subsequent studies, the results of which will be published elsewhere in due time. 

## 4. Materials and Methods

### 4.1. General

Reagents and solvents were of commercial sources, in the highest degree of purity and were used as received. Water used for the preparation of the solution was doubly-distilled and filtered through a Millipore^®^ milli-Q membrane (Millipore Merck KGaA, Burlington, MA, USA). UV/Vis spectra were recorded on a Varian Cary 100 Spectrophotometer (Santa Clara, CA, USA) with cell holder set at 298 K. CD spectra were performed on a JASCO J-1500 (JASCO-Europe Corporation, Italy), equipped with a cell holder set at 298 K, and purged with ultra-pure nitrogen gas. Linear Dichroism contribution (LD) has been found to be, in all cases lower than 0.0004 DOD units. Microstructural analysis of porphyrin aggregates was carried out by using a Field Emission Scanning Electron Microscope FE-SEM, SUPRATM 35, Carl Zeiss SMT, Oberkochen, Germany. Samples were carefully taken from the solution by the aid of a micropipette (Gilson^®^ P20), filtered through a Nylon set, rapidly washed with a 25/75 (*v*:*v*) ethanol/water mixture and transferred on a previously cleaned aluminum stub (MeOH; then N_2_ flow). This procedure allows for the removal of most of the amines excess from the mother solution. Extensive washing procedure could not be possible, due to solubilization of the solid precipitate (dissolution equilibrium of the macrocycles in monomeric form). This finding, unfortunately, hampered an exact evaluation of the porphyrin/amine ratio by, for example, 1H-NMR spectrometry. The samples layered on glass slides were examined with a Zeiss fluorescence microscope (Axio Scope.A1) equipped with a mercury-vapor short-arc-lamp HB = 50 W/AC at 460 nm emission wavelength, with a 63× (or 100×) objective, and digital images were acquired by an Axiom 503 mono (Zeiss, Oberkochen, Germany) and analyzed with Axiovision software (Zeiss). Glass slides were cleaned by treatment with piranha solution, then thoroughly rinsed, in this order with distilled water, methanol, and finally dried by nitrogen flush. Sonication of the prepared solutions was performed by ultrasound thermo-bath Fisher Scientific FB 15,047 (Italy), 90 W power supply, ultrasonic frequency of 37 kHz.

### 4.2. Synthesis of Porphyrin Derivatives

Synthesis of the porphyrins was accomplished by following procedures reported by our group [8,32], which relies on straightforward procedures used in peptide chemistry. As a first stage, the 5-(4-carboxyphenyl)-10,15,20-triphenylporphyrin (H_2_pCTPP) was reacted with (*L*)- or (*D*)-proline *tert*-butyl ester using the EDCl/HOBT coupling reagents, providing the corresponding prolinated derivatives which were subsequently hydrolyzed in a TFA/CH_2_Cl_2_ mixture to give the amphiphilic compounds (*L*)H_2_P(-) and (*D*)H_2_P(-). The three porphyrin free bases were further metalated with an excess of zinc acetate in CHCl_3_/MeOH, affording the corresponding (*L*)ZnP(-), (*D*)ZnP(-), and ZnpCTPP(-) complexes. The reaction scheme is reported as Supplementary Material (Scheme S1). The enantiomeric purity of porphyrin samples was verified by chiral HPLC analysis [32]. 

### 4.3. Aggregation and Kinetic Studies

All the spectroscopic studies were carried out at 298 K. Solutions suitable for the aggregation studies were prepared as follows. Porphyrin stock solutions in ethanol (ca. 10^−4^ M concentration) were gently warmed, then briefly sonicated and finally filtered through a 0.22 μm Nylon^®^ membrane (Hahnemüle Albet^®^ Syringe Filters) prior to use. These precautions were taken to avoid uncontrolled nucleation of porphyrin protoaggregates that would affect the reproducibility of the experiments. Checking of the effective concentration was made by UV-Vis spectroscopy in pure ethanol (Soret band intensity of the porphyrins in monomeric form; ε = 4.51 × 10^5^ M^−1^ cm^−1^). Aggregation of the investigated porphyrins was carried out in aqueous ethanol solutions (EtOH/H_2_O 25:75 *v*:*v*; 298 K) at 5.0 and 10 μM concentration, by strictly following a “porphyrin first” protocol. Previously published studies, carried out by our group and by others, pointed out in fact the strong dependence of the aggregation process on the experimental conditions, such as the order in which the reagents are mixed [42]. Namely, a proper aliquot of a stock solution of porphyrin (10 to 100 μL) was added to the required amount of ethanol (final volume of 1.0 mL) in an 8 mL glass vial, and briefly sonicated. To this solution the required amount of amine was then added. Finally, the solution was quenched with a slow addition of 3.0 mL of water, again under sonication, to give 4.0 mL of resulting solution with 25% *v*:*v* solvent proportion, with the required porphyrin and amine concentration. A ca. 2.5 mL portion was transferred into a quartz cuvette and the relative spectra were then acquired at different times to follow the temporal evolution of the systems. This second sonication step appears to be of crucial importance for a good reproducibility of the results, that depends on the homogeneity of the morphology of the initially formed porphyrin clusters, which would be detrimentally affected by the unavoidable occurrence of both gradients of concentration and solvent polarity changes during the mixing with water [11]. The porphyrin stock solutions should be used within two weeks from preparation, to ensure optimal reproducibility of the results. UV-Vis and CD spectra were recorded with time, in order to acquire information on the progress of the self-assembly process. 

## Supplementary Materials

The following are available online at https://www.mdpi.com/1422-0067/21/22/8557/s1, Scheme S1: Scheme of the synthesis of the compounds used in the work, Figure S1: CD spectra of equilibrium solutions of (*D*)ZnP(-) and (*L*)ZnP(-) in the presence of (*R*)-1-phenyl-ethanamine and (*S*)-1-phenyl-ethanamine, Figure S2: UV-Vis and CD spectra of the aggregates of (*D*)ZnP(-) in the presence of achiral benzylamine, Figure S3: UV-Vis spectral variations with time of (*L*)ZnP(-) in the presence of (*R*)-1-phenyl-ethanamine, and corresponding kinetic plot, Figure S4: UV-Vis and CD spectral variations with time of (*L*)ZnP(-) (10 μM) in the presence of (*R*)-1-phenyl-ethanamine (1.0 × 10−3 M), Figure S5: SEM topographies of precipitates from 10 μM equilibrium solutions of (*D*)ZnP(-) (A) and (*L*)ZnP(-) (B) in the presence of (*S*)-1-phenylethanamine, Figure S6: Microscope transmission and fluorescence emission images of drop casted equilibrium solution on glass of (*D*)ZnP(-)@(*R*)-1-phenylethanamine solution.
