# Supplementary material for: Tunable Supramolecular Chirogenesis in the Self-Assembling of Amphiphilic Porphyrin Triggered by Chiral Amines
Source: Int J Mol Sci. 2020 Nov 13;21(22):8557. doi: 10.3390/ijms21228557 (PMC7698035; doi:10.3390/ijms21228557)
Supplement: Supplementary file 1 [file ijms-21-08557-s001.pdf]

Tunable Supramolecular Chirogenesis in the Self-Assembling of Amphiphilic Porphyrin-Amine systems.

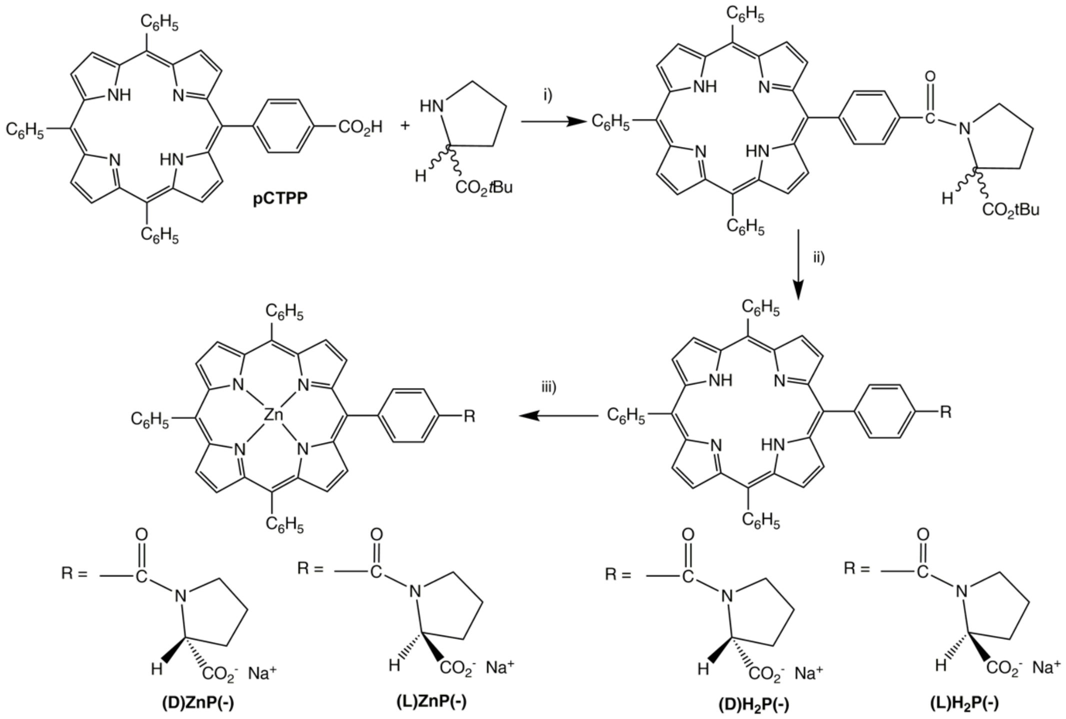

Scheme S1. i) EDCl, HOBT, dry CH_2Cl_2, 0°C, 1h; then RT, 48 h. ii) TFA/CH_2Cl_2 (2/3, v/v), 1.5 h, then aqueous $NaHCO_3$. iii) $Zn(OAc)_2·2H_2O$, $CHCl_3$/MeOH, RT, 1h. Metalation, and subsequent treatment of **pCTPP** ($Zn(OAc)_2·2H_2O$, $CHCl_3$/MeOH, rt, 1h; then aqueous $NaHCO_3$) afforded **ZnpCTPP(-)**. Spectroscopic and analyutical data were in full agreement to those reported in literature. See reference: Chouikrat, R., Champion, A., Vanderesse, R., Frochot, C., Mussaron, A. *J. Porphyrins Phthalocyanines*. 2014, **18**, 1-6.

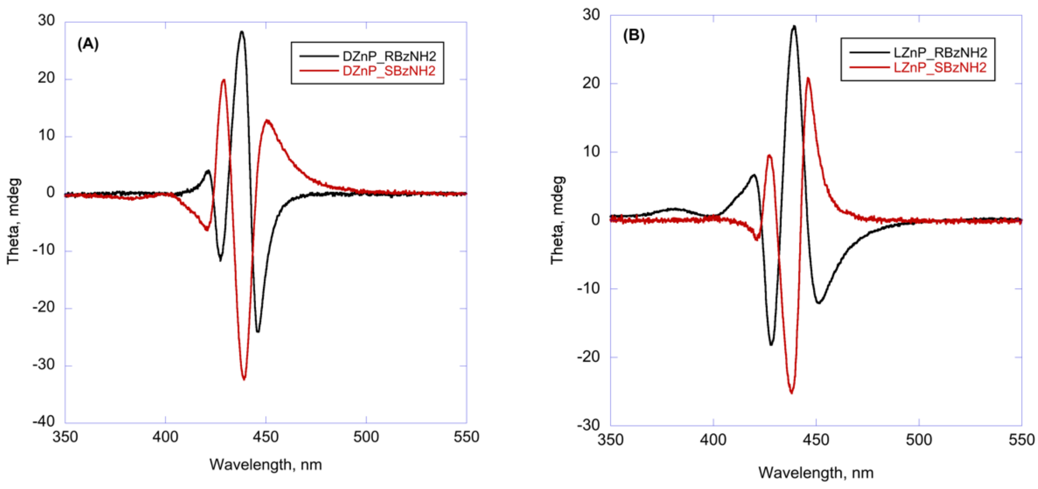

Figure S1. CD spectra of equilibrium solutions (5 μM; EtOH/H_2O 25/75 v:v; 298 K) of: (A) **(D)ZnP(-)** in the presence of **(R)-1-phenyl-ethanamine** (black trace), **(S)-1-phenyl-ethanamine** (red trace). (B) **(L)ZnP(-)** in the presence of **(R)-1-phenyl-ethanamine** (black trace), **(S)-1-phenyl-etnanamine** (red trace).

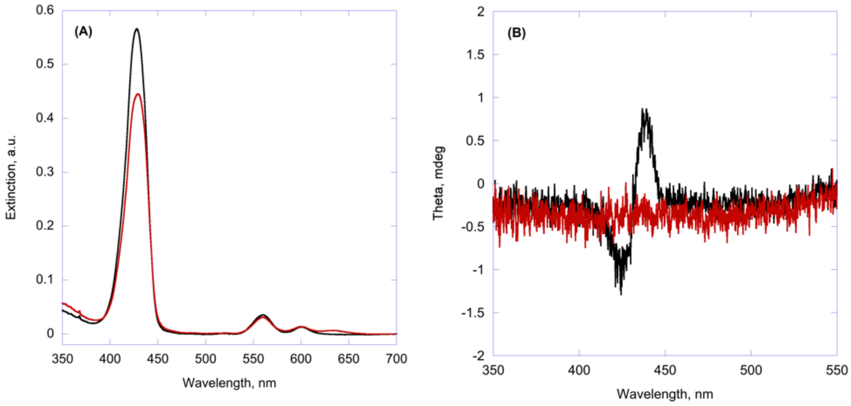

Figure S2. UV-Vis (A) and CD spectra (B) of the aggregates of **(D)ZnP(-)** (5 μM; EtOH/H_2O 25/75 v:v; 298 K) in the presence of achiral benzylamine (5.0 x 10^{-4} M) at t = 0 (black traces), and at equilibrium (red traces).

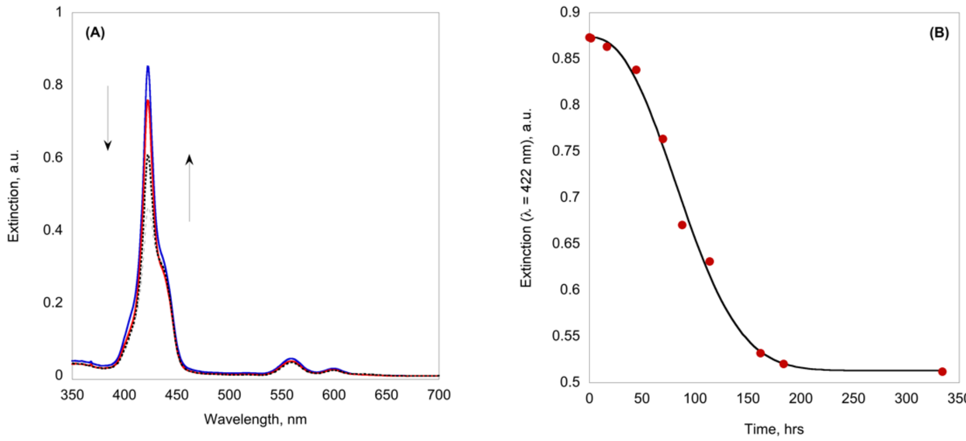

Figure S3. (A) UV-Vis spectral variations with time of **(L)ZnP(-)** (5 μM; EtOH/H_2O 25/75 v:v; 298 K) in the presence of (R)-1-phenyl-ethanamine (5.0 x 10^{-4} M). (B) Corresponding kinetic plot (λ = 422 nm).

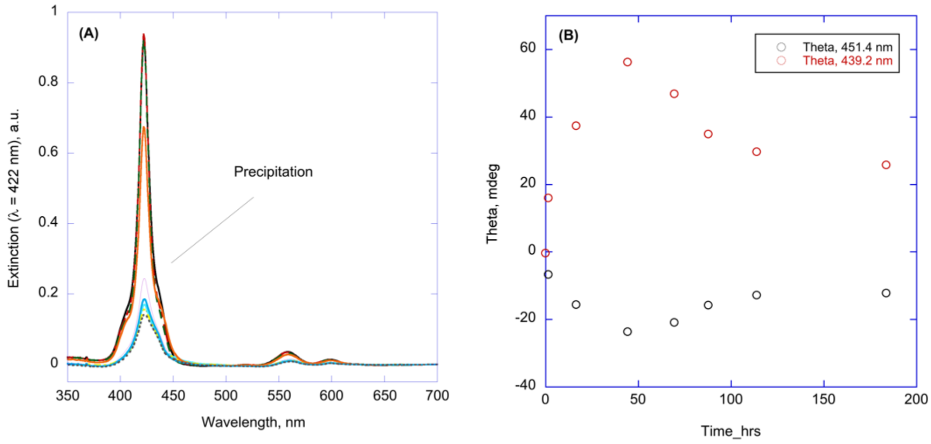

Figure S4. (A) UV-Vis spectral variations with time of **(L)ZnP(-)** (10 μM; EtOH/H_2O 25/75 v:v; 298 K) in the presence of (R)-1-phenyl-ethanamine (1.0 x 10^{-3} M). (B) Corresponding CD plot at λ = 451 nm (black circles) and λ = 439 nm (red circles).

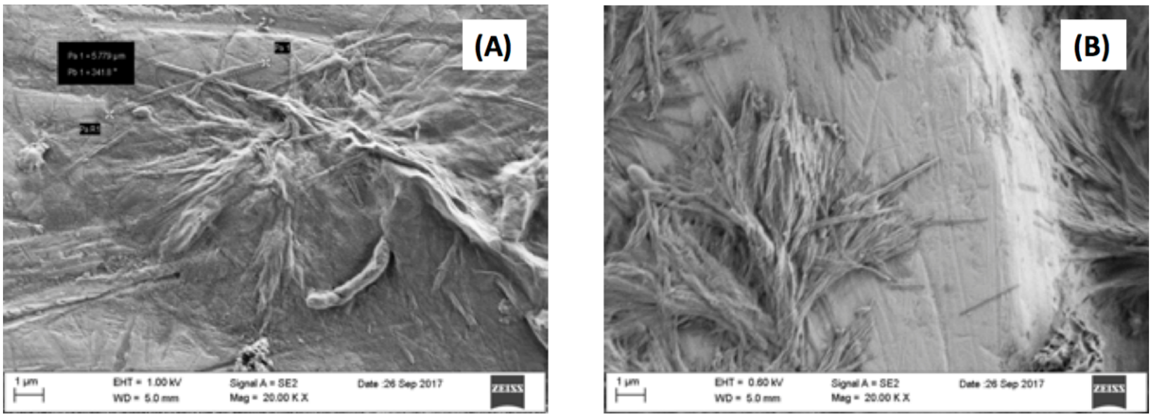

Figure S5. SEM topographies of precipitates from 10 μM equilibrium solutions of **(*D*)ZnP(-)** (A) and **(*L*)ZnP(-)** (B) in the presence of **(*S*)-1-phenylethanamine**.

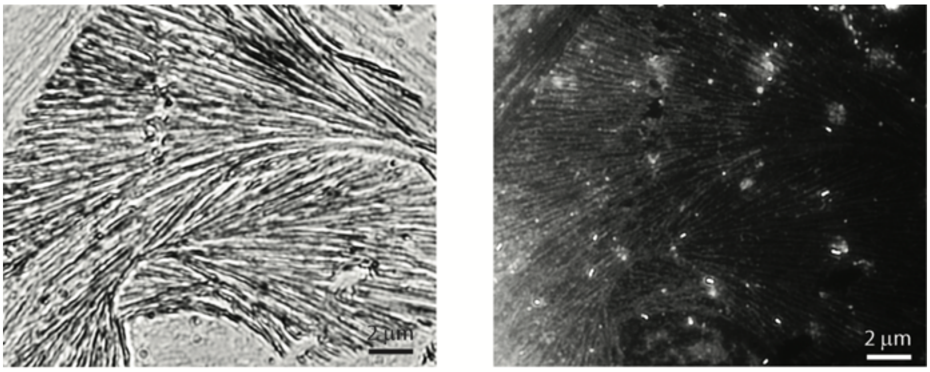

(a) (b)

Figure S6. (A) Microscope transmission images of drop casted equilibrium solution on glass of (A) **(*D*)ZnP(-)@(*R*)-1-phenylethanamine** solution, and corresponding fluorescence emission image, evidencing the quenching of fluorescence of the sample.

Table 1. Spectroscopic parameters for the CD spectra of porphyrin aggregates (EtOH/H_2O 25/75 % (v:v) at 5.0 μM, 298 K) in the presence of 5.0 x 10^{-4} M of (R)- or (S)-1-phenylethanamine (amine). The uncertainties of the values are within ± 1 nm.

	Crossover wavelength, nm	
**Porphyrin**	B_H	B_J
(D)ZnP(-) [(a)]	420 (+ /-)	442 (-/+)
(L)ZnP(-) [(a)]	421 (-/+)	441 (+/-)
(D)ZnP(-)@(R)-amine	423 (+/-)	431; 443 (-/+/-)
(D)ZnP(-)@(S)-amine	423 [(b)]	432; 444 (+/-/+)
(L)ZnP(-)@(R)-amine	424 (-/+)	433; 446 (-/+/-)
(L)ZnP(-)@(S)-amine	424 [(b)]	431; 442 (+/-/+)
ZnpCTPP(-)@(R)-amine	-	433 nm (-/+)
ZnpCTPP(-)@(S)-amine	-	433 nm (+/-)

(a) Experiment carried out in the absence of amine; see reference 29. [(b)] The sign of the couplet of the B_H band could not be ascertained with accuracy due to partial overlap to the more intense B_J transition.
